# Unhappy While Depressed: Examining the Dimensionality, Reliability and Validity of the Subjective Happiness Scale in a Spanish Sample of Patients with Depressive Disorders

**DOI:** 10.3390/ijerph182010964

**Published:** 2021-10-19

**Authors:** Albert Feliu-Soler, Javier de Diego-Adeliño, Juan V. Luciano, Ioseba Iraurgi, Carlo Alemany, Dolors Puigdemont, Víctor Pérez, Maria J. Portella, Joan Trujols

**Affiliations:** 1 Department of Clinical & Health Psychology, Autonomous University of Barcelona (UAB), 08193 Bellaterra, Spain; albert.feliu@uab.cat; 2 Teaching, Research & Innovation Unit, Parc Sanitari Sant Joan de Déu, 08830 Sant Boi de Llobregat, Spain; 3 Servei de Psiquiatria, Hospital de la Santa Creu i Sant Pau, Institut d’Investigació Biomèdica Sant Pau (IIB Sant Pau), 08025 Barcelona, Spain; fdiego@santpau.cat (J.d.D.-A.); calemany@santpau.cat (C.A.); mpuigdemont@santpau.cat (D.P.); mportella@santpau.cat (M.J.P.); 4 Centro de Investigación Biomédica en Red de Salud Mental (CIBERSAM), 28029 Madrid, Spain; vperezsola@parcdesalutmar.cat; 5 DeustoPsych—Unidad de Investigación, Desarrollo e Innovación en Psicología y Salud, Universidad de Deusto, 48007 Bilbao, Spain; ioseba.iraurgi@deusto.es; 6 Institut de Neuropsiquiatria i Addiccions (INAD), Parc de Salut Mar, 08003 Barcelona, Spain; 7 Grup de Recerca en Psicologia Quantitativa (SGR 269), Unitat de Neurociència Quantitativa, Universitat de Barcelona, 08035 Barcelona, Spain

**Keywords:** happiness, depressive disorders, assessment, self-report, Subjective Happiness Scale, psychometrics

## Abstract

Despite the considerable amount of research evidence on the significant role of subjective happiness on mental health, there is no psychometric study of the Subjective Happiness Scale (SHS) in psychiatric samples. This study was aimed at exploring the psychometric properties of the SHS in a Spanish sample of patients with depressive disorders. Participants were 174 patients with a depressive disorder (70% diagnosed as major depressive disorder) who completed the SHS, the Quick Inventory of Depressive Symptomatology-Self Report (QIDS-SR_16_), and the EQ-5D Visual Analogue Scale (EQ-5D VAS). Depressive symptoms were also assessed by means of the 17-item Hamilton Depression Rating Scale (HDRS_17_) and the Clinical Global Impression-Severity (CGI-S) Scale. Dimensionality, internal consistency reliability, construct validity, and responsiveness to change of the SHS were examined. Confirmatory factor analysis replicated the original one-factor structure of the scale. The SHS exhibited good-to-excellent results for internal consistency (α = 0.83) and for convergent [EQ-5D VAS (r = 0.71)] and divergent [QIDS-SR_16_ (r = −0.72), HDRS_17_ (r = −0.60) and CGI-S (r = −0.61)] construct validity. The ability of the SHS to differentiate between depression severity levels as well as its responsiveness to clinical change were both highly satisfactory (*p* < 0.001 in both cases). The SHS retained the soundness of psychometric properties showed in non-clinical samples in a sample of patients with depressive disorders, which supports its use as a reliable and valid outcome measure in the treatment of such disorders.

## 1. Introduction

The achievement of happiness has been identified as an ultimate goal for most societies [1,2]. Happiness can be understood as the mirror of subjective well-being [3] and is a construct determined by the interaction of genetic, emotional and cognitive factors [4,5]. Although people widely differ in the sources of their personal happiness, there is a common understanding about what happiness means and to what degree it has been accomplished [6]. In this line, we can easily identify individuals who seem consistently happy, even in the face of difficulty, and others who are chronically unhappy, despite apparently living in favorable circumstances [6,7]. Although it seems to be a relatively stable construct [6], there is mounting evidence regarding the possibility to be modified, not simply because of life events and other personal circumstances but also as a result of psychological interventions [2,8,9,10,11,12].

Subjective happiness has been found to be associated (negative correlations of small-to-moderate effect sizes) with depressive symptomatology in the general population [13,14,15], health professionals [16] and students [17]. In this regard, positive affective experience—which is associated with happiness—has been described as an important component of mental health and its absence has been argued to be an inherent feature of depression [18,19]. In a meta-analytic study [20] happiness was found to be related to greater quality of life, more successful social relationships, better mental and physical health, more successful careers, better conflict resolution skills, among many other positive outcomes. Furthermore, this study concluded that higher happiness levels could precede many of these outcomes suggesting some sort of causality [20].

Mental health disorders seem to be more strongly associated with happiness than physical health conditions [21]. Alcohol and drug disorders, anxiety, depression or other mental illnesses have stronger effects on subjective well-being and happiness than physical health problems [22]. Particularly, patients suffering from other psychiatric disorders such as schizophrenia [23,24], or attention deficit and hyperactivity disorder [25] showed significantly lower levels of happiness or subjective well-being than healthy controls, and depressive symptoms are a common mediator of these associations. This is also true for patients diagnosed with generalized anxiety disorder, in which having a lifetime history of major depressive disorder is one of the significant factors related to lack of happiness and well-being [26]. Having had a depressive disorder during the last 6–12 months is also an independent predictor of reduced happiness, especially in persons experiencing more severe symptoms [27,28]. Levels of happiness have been also associated with suicide rates and hospital discharge rates for mental disorders in ecological studies [29]. Unhappiest people, in fact, seem to be at higher risk of committing suicide during their lives [30]. Happiness and psychopathology in psychiatric disorders seem to be closely correlated, although they are not completely mutually exclusive and yet there are many patients with common mental disorders (including depression) that reported moderately high levels of happiness or constructs closely related to it [28,31].

Besides, individuals reporting high trait positive affect also present a lower chance of suffering depression [20] as well as social phobia or anxiety [32]. In turn, individuals with greater dispositional optimism show higher levels of self-reported vitality and mental health [33] and lower levels of depression [20,34].

Furthermore, a series of studies has shown that unhappier people are more sensitive to negative feedback from their peers, as well as more emotionally affected, and they show greater deteriorations in their mood, self-confidence and perceived competence [35]. This increased sensitivity overlaps with negative cognitive and attributional styles in depression, thus potentially contributing to the etiology and maintenance of depressive episodes [35]. Coherently, positive cognitions and emotions—intrinsic to happiness—have been shown to soften automatic negative thoughts and so to provide a protective effect on stress and depression [36]. Nevertheless, it is not clear whether a reduced experience of happiness is really a causal factor to develop depression or whether the presence of a previous depressive syndrome or some of their well-known related risk factors leads to diminished levels of happiness. It has been reported that personality traits like extraversion or neuroticism attenuate the relationship between baseline depression and future happiness in a remarkable longitudinal study [28]. Therefore, happiness could be an interesting complementary risk marker and outcome measure for mood disorders.

During the last five decades, there has been a considerable amount of research on subjective happiness (usually defined as subjective well-being), on its determinants or sources, and on how it can predict or be related to multiple variables such as personal success, physical and mental health. Along with this research, many instruments have been developed to measure it in a reliable, valid and sensitive way. In this regard, most studies used self-reports since happiness is a personal, subjective experience [3]. Most operationalizations of the construct were oriented to specifically assess high positive affect, low negative affect or satisfaction with life (e.g., the Affect Balance Scale [37], the Delighted-Terrible Scale [38], and the Satisfaction with Life Scale [39]). Given the limited scope of previous measurements, Lyubomirsky and Lepper [6] developed a wider measure of subjective hedonic well-being—the Subjective Happiness Scale (SHS)—to assess global subjective happiness. The SHS asks for perceived levels of happiness and the interpretation of own happiness in relation to others. The instrument was initially composed of 13 items but, after an item pruning process, only four non-redundant items loading on a single factor remained in its final version [6].

Nowadays, the SHS is one of the most used instruments for measuring global happiness and it has shown—in diverse and large samples of students and community adults—high internal consistency, good-to-excellent test-retest reliability, a unidimensional structure, and satisfactory convergent (with moderate correlations with other well-being variables) and discriminant validities, not only in its original version [6] but also in its subsequent linguistic adaptations. The original English version has been translated and cross-culturally validated in several languages: Arabic [40,41], French [41,42], Chinese [43,44], German [45], Hungarian [46], Italian [47], Japanese [48], Malay [49], Portuguese [50,51], Russian [6], Serbian [52], Spanish [13,14,53], Tagalog [45] and Turkish [54]. Despite this large number of linguistic adaptations of the SHS as well as the considerable amount of research evidence on the significant role of subjective happiness on mental health (e.g., [20]), there is, as far as we know, no psychometric study of this scale in clinical psychiatric samples.

The present study is aimed at exploring the psychometric qualities of the SHS in a Spanish clinical sample of patients with depressive disorders. The following characteristics of the scale are examined: dimensionality, internal consistency reliability, construct validity (both convergent and divergent), and responsiveness to clinical change. We hypothesize that the psychometric properties of the SHS in a Spanish sample of patients with depressive disorders will be similar to those observed with the original and Spanish versions of the instrument in non-clinical samples. Regarding validity specifically, the following three hypotheses are particularly explored: (a) Scores in the SHS will positively correlate (moderate-to-strong effect size) with a visual analog scale addressing overall health-related quality of life (HRQoL). (b) Scores in the SHS will be significantly associated with two instruments measuring dissimilar constructs such as depression (with a negative correlation of moderate-to-strong effect size) and social support (positive correlation with a small-to-moderate effect size). (c) Scores in the SHS will account for a significant proportion of variance of the measure of HRQoL above and beyond the variance accounted for by a self-reported measure of depression. With regard to sensitivity to change of the scale, the following two hypotheses are specifically addressed: (a) Patients with greater clinical improvement (i.e., higher change scores on the CGI-S during antidepressant treatment) will show a greater increase in SHS scores. (b) Effect size of the changes on the SHS will be of the same magnitude as those observed in three instruments measuring depressive symptoms.

## 2. Method

This is an essentially cross-sectional study, carried out in the Department of Psychiatry of Hospital de la Santa Creu i Sant Pau (a public university hospital in Barcelona, Catalonia), with a longitudinal component for the scale responsiveness to change assessment (in which a subset of participants—approximately 15% of the sample—were followed up and re-evaluated three or more weeks after, under conditions of routine clinical practice).

### 2.1. Participants

The sample consisted of 174 patients of Spanish origin (65.3% women), with a mean age of 50.3 (SD = 14.9) years. Demographic and clinical details of the sample can be found elsewhere [55]. In brief, fifty per cent of included participants were married or cohabiting with a partner; thirty per cent were single; and the rest were separated, divorced or widowed. Almost four-fifths had secondary school level or higher, while only three per cent of participants had no formal education. In terms of daily activity, more than a half were active (market work, housework and/or students); the remainder were unemployed (7%), on sick leave (21%) or retired (18.5%). Further information regarding the sample’s characteristics is available in Table S1 as Supplementary Material. To be included in the study, all patients suffered from some kind of depressive disorder, i.e., a mood disorder according to DSM-IV (70.1% diagnosed as major depressive disorder; 12% as bipolar I disorder, last episode depressed; and 7.2% as dysthymic disorder) or from an adjustment disorder with depressed mood (10.8%). Mostly, they received treatment at the outpatient clinic of the hospital (84.1%) and were visited by trained clinicians—with extensive experience in research and clinical practice—who determined diagnoses through unstructured clinical interviews. The only exclusion criterion was not being able to command Spanish language (verbal comprehension, oral expression and reading competency). For the full sample, the mean score of the 17-item Hamilton Depression Rating Scale (HDRS_17_) [56,57] was 12.7 (SD = 8.8; range: 0–33) and of the Quick Inventory of Depressive Symptomatology-Self Report (QIDS-SR_16_) [55,58,59,60] 11.7 (SD = 6.7; range: 0–26), values that correspond to mild-to-moderate level of depressive symptoms according to the commonly-accepted correspondences between score ranges and severity levels [58,59,61,62]. During the study, all patients received their usual pharmacological and/or psychological treatments, regardless if recently prescribed or long-standing.

### 2.2. Measures

#### 2.2.1. Subjective Happiness Scale (SHS)

The Spanish version of the SHS used in the present study is the one validated by Extremera and Fernández-Berrocal [13]. It was developed following a standard translation/back-translation procedure (one forward translation and one backward translation), and validated in a large sample of students and community adults. In that validation study, the Spanish version of the SHS showed a clear one-factor structure, adequate internal consistency (range of Cronbach’s α: 0.79–0.83), satisfactory convergent validity with satisfaction with life (r = 0.67), and adequate pieces of evidence of divergent validity with depressive symptomatology (r = −0.52) and trait anxiety (r = −0.60).

#### 2.2.2. Depressive Symptomatology

Depressive symptoms were assessed by means of the QIDS-SR_16_ [55,58,59,60] and two interviewer-rated instruments: the HDRS_17_ [56,57] and the Clinical Global Impression-Severity (CGI-S) Scale [63,64]. In the present study, internal consistency reliability for both the QIDS-SR_16_ (α = 0.880, 95% CI: 0.851, 0.905) and the HDRS_17_ (α = 0.883, 95% CI: 0.855, 0.908) was good.

#### 2.2.3. Quality of Life and Social Support

In addition, participants were also assessed by means of two self-report instruments tapping into health-related quality of life and perceived social support: the EQ-5D Visual Analogue Scale (EQ-5D VAS) [65,66] and the Multidimensional Scale of Perceived Social Support (MSPSS) [67,68], respectively. MSPSS’s internal consistency reliability in the present sample was excellent (α = 0.927, 95% CI: 0.910, 0.942).

### 2.3. Procedure

Participants were asked to fill all self-reported scales in and to respond to clinician-rated instruments. A research assistant guided the participants through instructions for all self-reported instruments, answered their questions regarding self-reported scales completion, and stayed through the assessment session until all scales were filled out. All self-reported instruments were administered individually in a single session and without the presence of clinical staff. Scores on self-reported scales were blinded to clinicians who administered the other instruments to avoid contamination. Due to missing values within each measure (range of missing values: 0–11, median = 3.5), sample sizes varied minimally.

Responsiveness to change of SHS was evaluated after re-administration of this scale together with the QIDS-SR_16_, HDRS_17_ and CGI-S three or more weeks after (median = 31.0 days) to a subgroup of patients (*n* = 27) who started an antidepressant treatment in the near days of study commencement.

The study was carried out following the principles of the Declaration of Helsinki and subsequent revisions and approved by the Clinical Research Ethics Committee of the Hospital. All participants gave their written informed content after receiving extensive details of the study.

### 2.4. Data Analyses

The commonly found factor structure of the SHS (i.e., unidimensional) was tested by means of a confirmatory factor analysis (CFA) conducted using the maximum likelihood robust (MLR) estimation method. Goodness of fit was evaluated by means of the following indices: the chi-square statistic (χ^2^), the comparative fit index (CFI), the Tucker-Lewis index (TLI), and the root-mean-square error of approximation and its 90% confidence interval (RMSEA).

Internal consistency reliability was tested by means of Cronbach’s alpha. Corrected item-total correlations were computed to assess homogeneity. Construct validity was assessed by correlating the SHS with a measure of quality of life (EQ-5D VAS) for convergent, and with the instruments measuring dissimilar constructs such as depression severity (QIDS-SR_16_, HDRS_17_ and CGI-S) and social support (MSPSS) for divergent validity. Incremental convergent validity was examined by means of a hierarchical regression analysis with EQ-5D VAS as the dependent variable, entering QIDS-SR_16_ scores in the first step and SHS scores in step 2. The known-groups construct validity of the scale was examined by grouping patients upon depression severity (according to the CGI-S) and comparing such groups on SHS scores with a one-way analysis of variance (ANOVA). Responsiveness to change of the SHS was explored in two different strategies: (a) comparison of SHS change scores (difference between second administration score minus first administration one) between the three categories of clinical improvement derived from CGI-S change scores; (b) comparison of effect sizes of the change scores on the SHS, QIDS-SR_16_, HDRS_17_ and CGI-S.

Analyses were performed using either IBM SPSS Statistics version 25.0 (IBM Corp., Armonk, NY, USA) or Mplus software version 7.4 (Muthén and Muthén, Los Angeles, CA, USA). Goodness-of-fit indices for the CFA were appraised based on the cut-off values recommended by Hu and Bentler [69] and Mueller and Hancock [70] (values in parentheses denote goodness-of-fit standards): the goodness-of-fit index (GFI ≥ 0.90), the comparative fit index (CFI ≥ 0.90), and the root mean square error of approximation (RMSEA ≤ 0.08). Cronbach’s alpha was interpreted conforming to Cicchetti [71], taking into account the ranges of clinical significance, while Pearson correlation coefficients and partial eta-squared (η^2^_p_) indexes in agreement with Ferguson [72] using the ranges for effect size interpretation.

## 3. Results

### 3.1. Dimensionality

All goodness-of-fit indices for the original one-factor model pointed to an excellent model fit to the data: χ^2^(2) = 2.266, *p* = 0.322; CFI = 0.999; TLI = 0.996; RMSEA = 0.028, 90% CI: 0.000, 0.156. Standardized factor loadings (λ), as reported in Table 1, were statistically significant and fall within the fair-to-excellent range.

### 3.2. Internal Consistency Reliability and Homogeneity

The Cronbach’s alpha coefficient was 0.826 (95% CI: 0.779, 0.864), indicating good internal consistency reliability [71]. In fact, the removal of the SHS item #4 led to even a significant increase of the scale’s alpha coefficient value (Table 1). Furthermore, all corrected item-total correlations were above the minimum cut-off of 0.30 recommended by Nunnally and Bernstein [73], reflecting satisfactory scale homogeneity.

### 3.3. Convergent Construct Validity

As hypothesised, the correlation of SHS with the measure of quality of life (EQ-5D VAS, r = 0.714, *p* < 0.001) was significant, positive and in the moderate-to-strong range [72].

#### Incremental Convergent Validity

As also hypothesised, adding the SHS in a hierarchical regression model resulted in a statistically significant R-squared change value [R^2^ change = 0.048, F(1,165) = 22.806, *p* < 0.001], indicating that the SHS accounted for 4.8% of unique variance in EQ-5D VAS scores above and beyond the QIDS-SR_16_.

### 3.4. Divergent Construct Validity

As expected, significant negative correlations were found between the SHS and the three scales measuring depressive symptoms: QIDS-SR_16_, r = −0.724, *p* < 0.001; HDRS_17_, r = −0.601, *p* < 0.001; and CGI-S, r = −0.612, *p* < 0.001. These correlation values are indicative of a moderate-to-strong effect size [72]. Likewise, a significant positive correlation but small-to-moderate in magnitude was found between the SHS and the measure of social support (MSPSS, r = 0.362, *p* < 0.001).

### 3.5. Known-Groups Construct Validity

Table 2 displays the SHS mean scores’ distribution according to the depression severity levels as measured by the CGI-S. A highly significant main effect of depression severity emerged [F(5,161) = 22.28, *p* < 0.001], in which less severe patients had higher SHS scores, and more severe, lower scores. Post-hoc Scheffé tests showing significant differences between pairs of CGI-S groups are also shown in Table 2.

### 3.6. Responsiveness to Change

Table 3 displays the SHS change scores (ΔSHS) according to the categories of clinical improvement (ΔCGI-S). The one-way ANOVA revealed a highly significant effect of clinical improvement [F(2,24) = 8.08, *p* = 0.002], i.e., patients with greater clinical improvement also showed a greater increase in SHS scores. As can be seen in Table 3, all but one post-hoc Scheffé comparisons were statistically significant. In the same line, effect size of the change in SHS scores over time (η^2^_p_ = 0.592) was comparable and consistent with effect sizes of the change on the QIDS-SR_16_ (η^2^_p_ = 0.547), HDRS_17_ (η^2^_p_ = 0.630), and the CGI-S (η^2^_p_ = 0.525). All these effect sizes were in the moderate-to-strong range according to the guidelines by Ferguson [72].

## 4. Discussion

The present study extends previous research by reporting the dimensionality, reliability, and validity of the Subjective Happiness Scale (SHS) for the first time in a clinical sample of patients with depressive disorders. Our results also give support to the notion that depressive symptomatology in patients who are suffering from this kind of disorder is clearly associated with their perception of happiness and subjective well-being. Significant robust negative relationships were found between self-rated happiness and depression severity. Moreover, levels of happiness seem to exert an independent effect—beyond the contribution of clinical symptoms—on health-related quality of life of patients with depressive disorders.

The CFA showed that the Spanish version of the SHS has a unifactorial structure in a psychiatric clinical sample, a finding congruent with the original unidimensional factor structure of the scale [6], as well as with prior psychometric studies conducted in Spanish-speaking non-clinical samples [13,14,53].

Previous psychometric analyses of the scale in non-clinical samples already demonstrated satisfactory internal consistency reliabilities. Relative to those studies, our findings (α = 0.83) are in the upper half of the range of reported Cronbach’s alpha coefficients (from 0.65 [54] to 0.94 [6]), indicating good internal consistency. Corrected item-total correlations from our study are mostly satisfactory, suggesting adequate homogeneity of the items. It should be noted, however, that item #4 (i.e., *Some people are generally not very happy. Although they are not depressed, they never seem as happy as they might be. To what extent does this characterization describe you*?) showed a small-effect-sized corrected item-total coefficient (r = 0.373). This same item also showed the lowest factor loading in CFA (λ = 0.380). This last result is a common outcome in the dimensionality analysis of psychological instruments comprised of a mixture of positively and negatively phrased items. Positively phrased items robustly load on the hypothesized factor whereas this is not the case of negatively phrased items [74].

Similar psychometric performances regarding this item have previously been reported by other authors (e.g., [13,44]). Such non-optimal functioning of item #4 may be related, as said, to the fact that this one is the only negatively formulated item of the scale (i.e., it really assesses “unhappiness”), and so may have a lower factor loading in comparison to the other items on the scale (i.e., those positively formulated). Furthermore, some authors such as O’Connor et al. [75] suggest a problem of wording in this item, that is, some respondents can be confused when answering this item because it seems to ask for the experience of being simultaneously not depressed and not happy. Many individuals can answer this item appropriately on the basis of the context provided by the other previous three items, but many do not. In fact, according to O’Connor et al. [75], item #4 is not informative enough to improve the measurement of happiness and thus could be deleted without compromising the psychometric properties of the scale. In this line, removal of this item in our study led to a non-negligible increase of the internal consistency of the SHS (from a Cronbach’s α of 0.83 to 0.90).

Coherently with previous literature on subjective happiness and overall health [20], we found a substantial convergence of the SHS with health-related quality of life as measured by the VAS of the EQ-5D (r = 0.714). In this regard, happiness has shown to be positively related to quality of life and with a large variety of health outcomes in diverse clinical and non-clinical samples [20,76,77,78]. Additionally, our results suggest that subjective happiness may be a protective factor against the effects of depression on health-related quality of life in patients with depressive disorders because the SHS accounted for an additional 4.8% of unique variance in VAS EQ-5D scores over and above clinical symptoms. Comparable results have recently been reported in a study on patients with rheumatoid arthritis [79], where happiness (assessed with SHS) was found to mitigate the negative effect of disease impact on health-related quality of life. In this regard, it is worth bearing in mind that health has been defined as “a state of complete physical, mental and social well-being and not merely the absence of disease or infirmity” [80], so it deems necessary to assess both positive and negative mental health indicators to provide a more complete health-status characterization of clinical and non-clinical populations.

In addition, SHS construct validity was significantly supported by highly negative correlations (between −0.60 and −0.72) with three instruments measuring the severity of depressive symptoms. Similarly, it should be noted that the Spanish version of the SHS significantly correlated with the Beck Depression Inventory (r > 0.5) in two studies from general population samples [13,14]. In this respect, several studies using different self-reported measures of positive mental well-being/subjective happiness and negative mental well-being have also found negative and moderate correlations between them, suggesting that positive mental health and negative mental health seem to be two correlated but distinct constructs (e.g., [20,81]) coherently with the two continua model of mental illness and mental health [82]. Regarding also the construct validity of the SHS, the present study has additionally found a significant, but small-effect-sized, positive correlation between subjective happiness scores and social support. Previous cross-sectional and longitudinal studies have likewise reported significant small-sized associations between happiness and perceived or actual social support (see [20], for a review).

Likewise, the SHS also showed a satisfactory discriminant or known-groups validity: SHS scoring is able to distinguish between different levels of depression severity measured with the CGI-S (i.e., the less severe patients had higher SHS scores and the more severe, lower scores).

Finally, congruently with previous research by Lyubomirsky et al. [11] and Tay and Kuykendall [12] supporting the changeability of happiness over time, our findings suggest that subjective happiness not only varies temporally but also that these changes may partially reflect—or at least covariate with—different degrees of symptomatic improvement of depressive disorders (i.e., patients with greater clinical improvement showed a greater increase in SHS scores). In addition and further underpinning its satisfactory responsiveness to change, the effect size of the change in SHS scores over treatment almost mimics the effect size of clinical changes measured by three different tools (i.e., QIDS-SR_16_, HDRS_17_, and CGI-S). This attribute confers an added value to the SHS as a potential outcome measure in the treatment of depressive disorders. This is particularly important if we consider that depressed patients rate the presence of features of positive mental health as very important when it comes to defining remission from depression [83,84]. In fact, positive affect and broader well-being are the main targets of emerging forms of psychotherapy for depression such as well-being therapy [85] and adaptations of positive psychology interventions (e.g., [86]).

### 4.1. Limitations

This study has potential limitations that deserve to be acknowledged. The main issue is the generalizability of the findings to other samples from distinct geographic areas and/or with different demographics and clinical characteristics, as the study was conducted in a single hospital. In any case, the current results may likely be invariant in similar settings. A second potential limitation lies in the common method bias threat. This possibility—similarly applicable to most real-world cross-sectional research—cannot be ruled out in every analysis but it seems improbable in those analyses which include both self-reported and clinician-rated instruments. Another shortcoming is that psychiatric diagnoses were based on unstructured clinical interviews, which could undermine the validity of diagnoses. However, this is the common way to establish a diagnosis in the real-world clinical practice, and, in psychometric studies, inclusion criteria do not need to be as strict as in other kinds of studies. The sample size in responsiveness to change analyses may be somewhat limited according to current standards [87], but anyhow, our results reached statistical significance. When planning this type of analysis, future studies should also consider including a control group since observed temporal changes in SHS scores may not be solely the result of the intervention, but also the unintended consequence of an eventual poor test-retest reliability of the measuring instrument in a given study. In any case, the current results are those of a psychometric study in real-world clinical practice (i.e., a psychometric study not embedded within a randomized controlled trial). Finally, given that those frequencies in the highest levels of CGI-S (level 6 and 7) were very low, these two categories were merged into one category. The known-group analyses were therefore run with six categories of depression severity instead of seven, which could slightly change the original meaning of the scale. In any case, the findings reflected valid and useful information for the interpretation of psychometric characteristics of the SHS.

### 4.2. Relevance for Clinical Practice

Depression severity deeply impairs the experience of happiness and well-being of people. These are two closely related but independent constructs with many other variables moderating their relationship. Whether happiness is the whole aim and end of human existence, as Aristotle [88] said, or not can be somewhat controversial, but happiness seems to be an outstanding complementary dimension to be explored in patients suffering from depressive episodes, especially as part of individual’s recovery goals.

## 5. Conclusions

The SHS retains the soundness of psychometric properties showed in non-clinical samples in a sample of patients with depressive disorders. The findings reveal a solid one-factor structure, good internal reliability, and adequate evidence of validity and responsiveness to change, making the SHS an invaluable and brief-to-use tool to assess subjective happiness both in clinical settings and research.

## Figures and Tables

**Table 1:** Means, standard deviations, standardized factor loadings (λ), corrected item-total correlations and Cronbach’s alphas if item deleted for the SHS (*n* = 174).

SHS Items (Content)	Mean	Standard Deviation	λ	Corrected Item-Total Correlation	Cronbach’s Alpha If Item Deleted
1. Happy person, in general	3.59	1.73	0.917	0.799	0.712
2. Happy compared to peers	3.42	1.78	0.942	0.793	0.713
3. Generally very happy, enjoying life, getting the most out of everything	3.29	1.78	0.751	0.687	0.764
4. Generally not very happy, never seem as happy as one might be (reverse scored)	4.33	1.83	0.380	0.373	0.901
SHS Total	3.66	1.44			

**Table 2:** SHS mean scores’ distribution according to depression severity levels as measured by the CGI-S (*n* = 167).

	CGI-S = 1 (*n* = 38)	CGI-S = 2 (*n* = 28)	CGI-S = 3 (*n* = 22)	CGI-S = 4 (*n* = 42)	CGI-S = 5 (*n* = 21)	CGI-S = 6–7 (*n* = 16)	Scheffé Post-Hoc Comparisons
SHS	5.01 (1.01)	4.24 (1.19)	3.40 (1.24)	2.92 (0.89)	2.99 (1.19)	2.33 (1.50)	(1) > (3) ***, (1) > (4) ***, (1) > (5) ***, (1) > (6–7) ***, (2) > (4) ***, (2) > (5) *, (2) > (6–7) ***

Values represent mean (SD). * *p* < 0.05; *** *p* < 0.001.

**Table 3:** SHS change scores (ΔSHS) according to the categories of clinical improvement derived from the CGI-S change scores (ΔCGI-S) (*n* = 27).

	ΔCGI-S = 0 or −1 (*n* = 9)	ΔCGI-S = −2 or −3 (*n* = 14)	ΔCGI-S = −4 or −5 (*n* = 4)	Scheffé Post-Hoc Comparisons
ΔSHS	0.50 (0.94)	2.68 (1.45)	2.75 (1.70)	(0 or −1) < (−2 or −3) ** (0 or −1) < (−4 or −5) *

Values represent mean (SD). * *p* < 0.05; ** *p* < 0.01.

## Data Availability

The data presented in this study are available on request from the corresponding authors.

## Supplementary Materials

The following is available online at https://www.mdpi.com/article/10.3390/ijerph182010964/s1, Table S1: Socio-demographic and clinical characteristics of participants (*n* = 174).
