# Supplementary material for: Unhappy While Depressed: Examining the Dimensionality, Reliability and Validity of the Subjective Happiness Scale in a Spanish Sample of Patients with Depressive Disorders
Source: Int J Environ Res Public Health. 2021 Oct 19;18(20):10964. doi: 10.3390/ijerph182010964 (PMC8535987; doi:10.3390/ijerph182010964)
Supplement: Supplementary file 1 [file ijerph-18-10964-s001.zip › ijerph-1374048-supplementary.pdf]

Table S1. Socio-demographic and clinical characteristics of participants (*n* = 174).

Gender	
Male	34.68%
Female	65.32%
Age	50.31 (14.88)
Educational level	
No formal education	3.01%
Primary studies	19.28%
Secondary studies	32.53%
University studies	45.18%
Marital status	
Married or with a stable partner	50.89%
Single	30.18%
Separated or divorced	13.61%
Widowed	5.33%
Occupational status	
Market work and/or housework	49.40%
Student	4.16%
Unemployed	7.14%
On sick leave	20.83%
Retired	18.45%
Main diagnosis	
Major depressive disorder	70.07%
Bipolar I disorder, most recent episode depressed	11.98%
Dysthimic disorder	7.19%%
Adjustment disorder with depressed mood	10.78%
Treatment facility	
Outpatient	84.12%
Inpatient	15.88%
$HDRS_{17}$	12.68 (8.76)
CGI-S	3.19 (1.67)
$QIDS\text{-}SR_{16}$	11.67 (6.73)
EQ-5D VAS	52.56 (26.07)
MSPSS	63.39 (17.13)
SHS	3.66 (1.44)

Values are given as mean (SD) unless otherwise indicated. CGI-S: Clinical Global Impression-Severity; EQ-5D VAS: EQ-5D Visual Analogue Scale; $HDRS_{17}$: 17-item Hamilton Depression Rating Scale; MSPSS: Multidimensional Scale of Perceived Social Support; $QIDS\text{-}SR_{16}$: Quick Inventory of Depressive Symptomatology–Self-report; SHS: Subjective Happiness Scale.
